# Waves of Adipose Tissue Growth in the Genetically Obese Zucker Fatty Rat

**DOI:** 10.1371/journal.pone.0008197

**Published:** 2010-01-22

**Authors:** Jennifer MacKellar, Samuel W. Cushman, Vipul Periwal

**Affiliations:** 1 Diabetes Branch, National Institute of Diabetes and Digestive and Kidney Diseases, National Institutes of Health, Bethesda, Maryland, United States of America; 2 Laboratory of Biological Modeling, National Institute of Diabetes and Digestive and Kidney Diseases, National Institutes of Health, Bethesda, Maryland, United States of America; Mayo Clinic College of Medicine, United States of America

## Abstract

**Background:**

In mammals, calories ingested in excess of those used are stored primarily as fat in adipose tissue; consistent ingestion of excess calories requires an enlargement of the adipose tissue mass. Thus, a dysfunction in adipose tissue growth may be a key factor in insulin resistance due to imbalanced fat storage and disrupted insulin action. Adipose tissue growth requires the recruitment and then the development of adipose precursor cells, but little is known about these processes in vivo.

**Methodology:**

In this study, adipose cell-size probability distributions were measured in two Zucker fa/fa rats over a period of 151 and 163 days, from four weeks of age, using micro-biopsies to obtain subcutaneous (inguinal) fat tissue from the animals. These longitudinal probability distributions were analyzed to assess the probability of periodic phenomena.

**Conclusions:**

Adipose tissue growth in this strain of rat exhibits a striking temporal periodicity of approximately 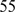 days. A simple model is proposed for the periodicity, with PPAR signaling driven by a deficit in lipid uptake capacity leading to the periodic recruitment of new adipocytes. This model predicts that the observed period will be diet-dependent.

## Introduction

The functional character of adipose tissue is of topical interest, given the marked increase in obesity that has been noted in much of the developed world. While the causes and consequences of this increase in obesity are the subject of debate, it is incontrovertible that obesity is an enlargement of adipose tissue to store excess energy intake. As such, the dynamic process by which adipose tissue grows is of great interest and potential medical significance.

While numerous studies of cell culture models of adipose cell differentiation and growth have provided many insights into the cellular events which occur during this process in vitro, little is known of this dynamic process in vivo. The literature suggests that an increase in adipose cell number is an early phenomenon in development [1], [2]. On the other hand, in obesity, an increase in cell size appears to predate the increase in cell number [3]. If different phases take place during adipose tissue growth, the signals for switching between phases are unknown. Adipose tissue obesity phenotypes are influenced by developmental stage, diet, and genetics, as well as by their interactions [4]–[13]. Much of this literature draws conclusions from studies of the mean sizes or other averaged characteristics of adipose cells. This may be misleading to some extent since it is now known that the cell-size distribution in adipose tissue is not a unimodal distribution [7], [8], [14]–[21]. Indeed, the functional characteristics of adipose tissue appear to depend on the finer details of the adipose cell-size distribution [18], [22].

The adipose tissue of young rats and mice grows with age through a combination of cell development, growth which involves stem cell differentiation, and a process through which small adipose cells gradually fill up with stored triglyceride. The mechanisms coordinating these processes are unknown, especially as fat storage continues increasing in such animals as male Sprague-Dawley rats, as well as other more specific genetic models of obesity where fat appears to grow indefinitely. An apparent dysfunction in this process has been reported to be associated with the insulin resistance of obesity and may even be the source of the insulin resistance that accompanies type II diabetes and other metabolic disorders [23]. Critical elements in the normal process, and likely sites of dysfunction in insulin resistance, are the communication networks within the growing adipose tissue fat depots and among adipose tissue, liver, and skeletal muscle. Preliminary experiments showed that the variability from animal to animal may be too large for time course data obtained from multiple animals to reveal the coordinated changes in adipose tissue. Therefore, we developed a surgical biopsy procedure for frequently sampling the inguinal fat depot so as to obtain a longitudinal time course of cell-size distribution measurements in individual animals.

Given the disparities [1]–[3] regarding the timelines of increasing adipose cell number and size, we asked if a temporal periodicity could be discerned in adipose tissue development, or if the growth of the tissue occurs monotonically as a continuous recruitment of precursor cells into the adipose lineage, followed by a steady growth of these cells to a maximal size. To address this issue, we obtained cell-size distributions of the inguinal fat pads in two male Zucker (fa/fa) fatty rats by micro-biopsies (Materials and Methods) over a period of 151 and 163 days respectively, starting at four weeks of age. The Zucker fatty rat is a well-characterized model of obesity and has fat depots that are large enough to allow repeated micro-biopsies to avoid between-animal variability. The tissue samples were collected at irregular intervals to avoid bias as we aimed to ascertain the (non-)existence of a temporal periodicity. We developed a Bayesian framework to select a period (including the case of no period at all) from these data. Applying the framework, we find that the development of adipose tissue proceeds in a periodic manner with a well-defined period.

## Results

### Adipose Cell-Size Distributions

**Figure 1:**
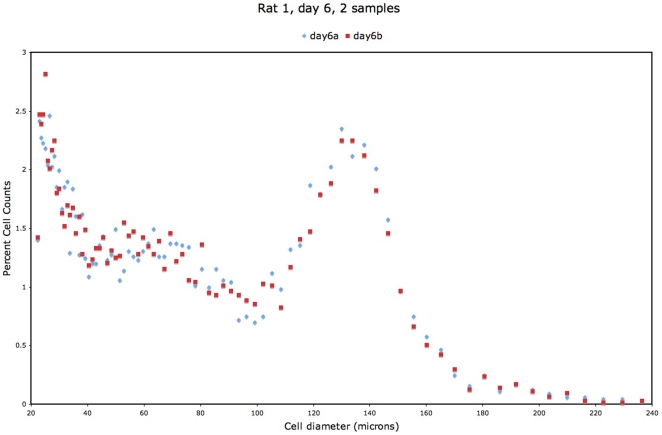
Cell-size data for rat 1, day 6.

**Figure 2:**
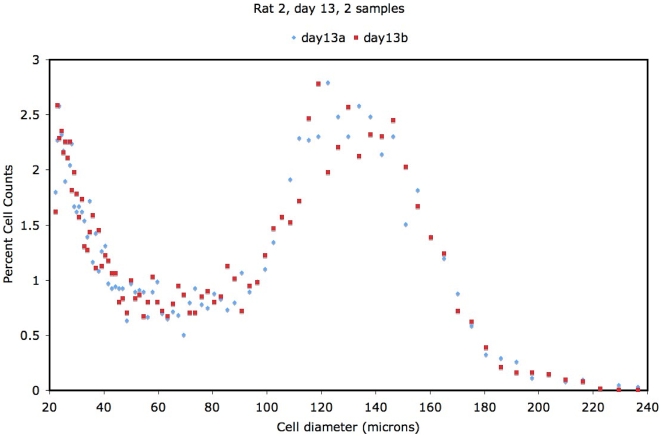
Cell-size data for rat 2, day 13.

**Figure 3:**
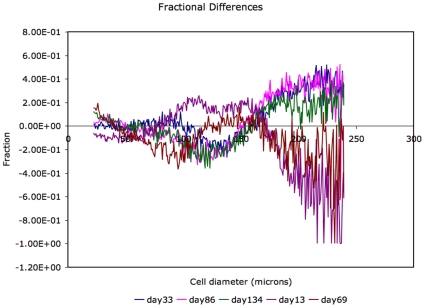
Fractional differences of adipose cell-size probability distributions (day 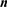 - mean)/mean as a function of cell diameter.

**Figure 4:**
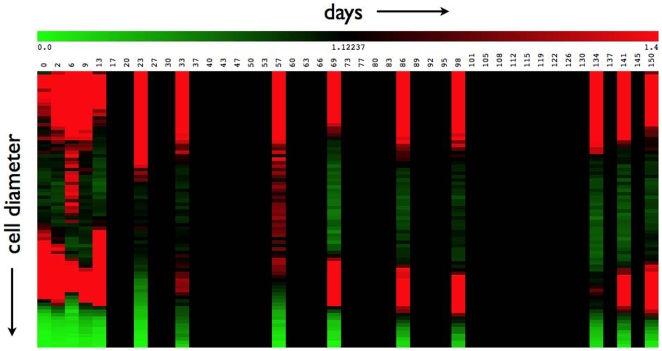
Heat-map showing data and gaps in data over the entire time-course for rat 1. The 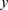
axis corresponds to cell diameter (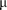
m, increasing towards negative 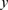) and the 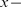
axis corresponds to time (days).

Representative adipose cell-size distributions (see Materials and Methods) for rat 1, day 6 are shown in Fig. 1, and for rat 2, day 13 are shown in Fig. 2. These demonstrate the consistency of the cell-size counts by this procedure. The bimodal form of the distribution is consistent with previous findings [7], [8], [14]–[21]. We define fractional differences relative to the mean adipose cell-size distribution over all time-points as follows: fract
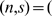
day
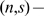
mean
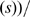
mean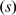, where 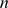 is the day of the experiment, 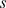 is the cell-size diameter, day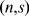 is the percentage of cells in the diameter bin 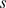 at day 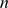, and mean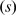 is the average cell-size distribution over all experimental days. Fig. 3 shows the similarity of fractional differences of adipose cell-size distributions at days 33, 86 and 134. Fig. 4 gives a global view of the entire data set to show evident dynamics and the limited data available to probe the dynamics.

### Log-Likelihood of Models

**Figure 5:**
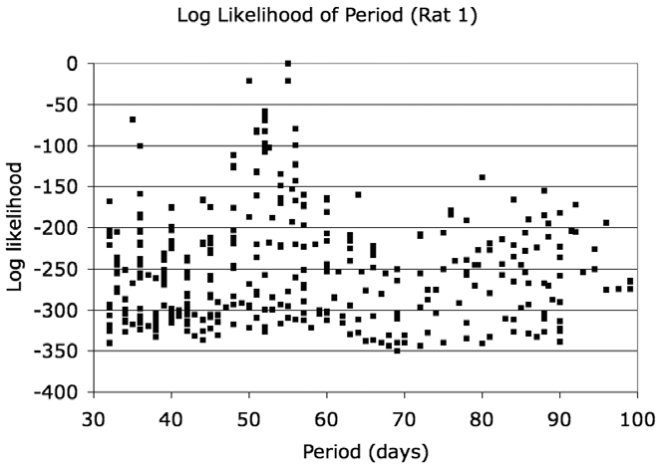
Log-likelihood of models for rat 1.

**Figure 6:**
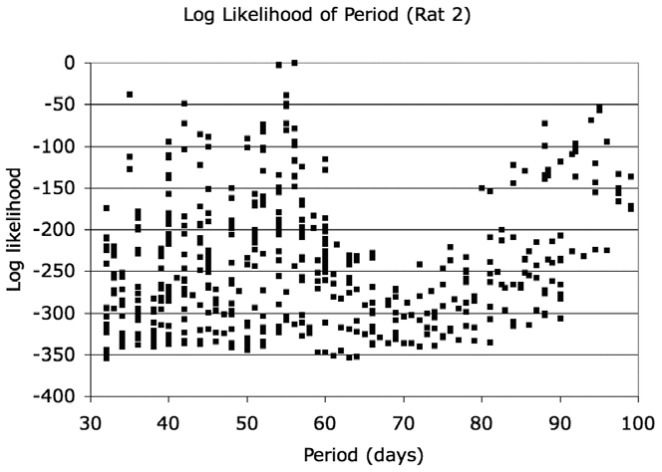
Log-likelihood of models for rat 2.

**Figure 7:**
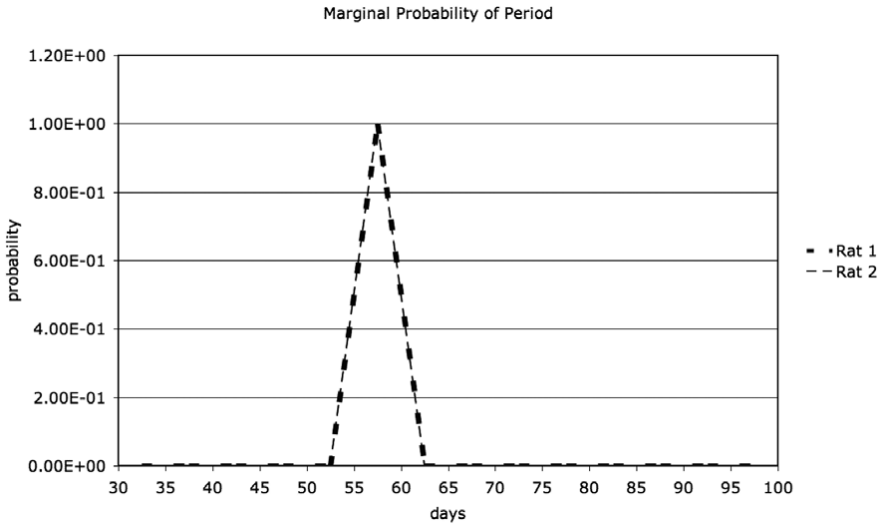
Probability of period as a function of period, marginalizing over all models (period-bin-size, bin number, phase) in 5-day intervals.

Since the data for rat 1 and rat 2 differ in the additional two time points available for rat 2 (days 156 and 162), the models evaluated for the two rats differ, with a larger number of models evaluated for rat 2. For rat 1, the most likely model had a period of 55 days and grouped the data into 5 bins of 11 days duration each. For rat 2, the most likely model had a period of 56 days and grouped the data into 7 bins of 8 days duration each. The phase for the most likely model for rat 1 was 4 days, and that for rat 2 was 7 days. The log-likelihood distributions for all models for rat 1 and rat 2 are shown in Fig. 5 and Fig 6, respectively. For rat 1, a log-likelihood difference of 20.8 is observed between the best-performing model and the next best model, which has a period of 50 days and 5 bins. For rat 2, the gap is more modest (3.5) between the best 3 models (56, 54 and 54 day periods), but the log-likelihood gap to the fourth best model (35–day period) is 38. The model of no periodicity had a log-likelihood below the worst periodic model for either animal and is not on the scale shown in Fig. 5 and Fig 6 for either animal. Marginalizing over models in 5–day wide intervals of periods, we show the most likely period in Fig. 7. To within the precision of the interval duration (5 days), the two animals have coincident predicted periods of adipose tissue development.

**Figure 8:**
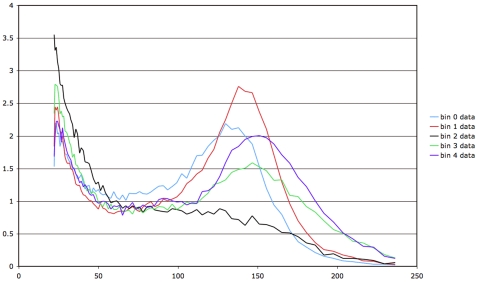
Cell-size probability distributions in each period-bin (from 0 through 4) for rat 1 for the most likely model, 5 bins of 11 days each.

**Figure 9:**
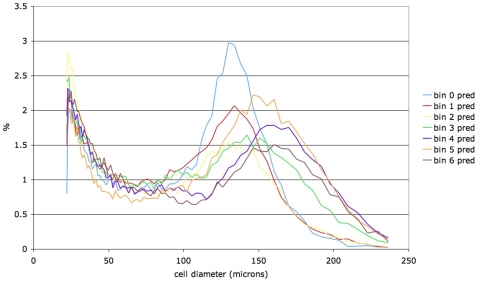
Cell-size probability distributions in each period-bin (from 0 through 6) for rat 2 for the most likely model, 7 bins of 8 days each.

Our method allows us to infer the progression of adipose cell-size distributions during the period, to within the precision of the duration of each bin. The period alone does not determine the most likely model, since there are numerous models with periods around 55 days with low likelihood. These models have bin sizes or phases that do not accurately capture the progression of cell-size distributions during a period. Cell-size distributions for each period-bin for the best performing models are summarized for rat 1 in Fig. 8 and for rat 2 in Fig. 9.

## Discussion

The adipose organ is central to energy homeostasis and therefore to animal survival. The dynamics of its growth and development impacts the fitness of the whole organism. For mammals, fat must be consumed or stored, suggesting teleologically that a capacity to excrete excess fat has not been necessary for survival. As in the case of another organ of metabolic importance, the liver, the adipose organ must, in certain circumstances, be capable of expanding to meet the demand for increased storage.

In the present study, we have found that rat adipose tissue undergoes periodic expansion, suggesting that a feedback mechanism is triggered when the capacity for lipid uptake is less than the flux of lipid needing storage. Thus, when continuing hypertrophy is incapable of storing lipid, perhaps due to characteristics of large adipocytes, hyperplasia may be triggered to provide additional storage [3] and thereby prevent lipotoxicity. Once new adipocytes have been recruited, the lipid uptake capacity may be greater than the lipid flux needing storage, and this may put an end to hyperplasia. Thus, an alternation between hypertrophy and hyperplasia may lead to oscillations, albeit only until the replicative capacity of adipocyte precursors is exhausted. [15] carried out a quantitative comparison of the relative roles of hypertrophy and hyperplasia, and their interplay with diet and genetics.

**Figure 10:**
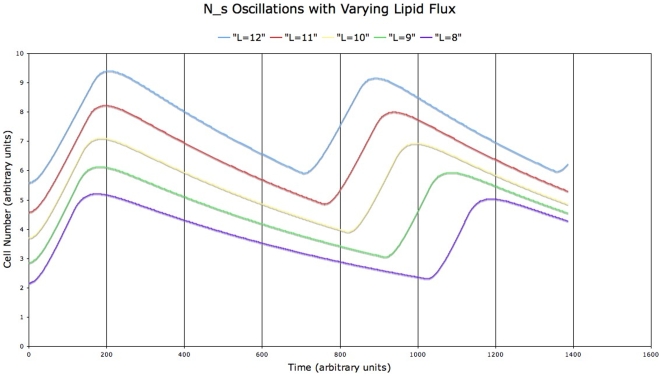
Periodic small adipocyte recruitment predicted by a simple model of adipocyte proliferation driven by lipid flux vs. lipid uptake capacity. The period is longer for lower lipid flux, 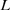.

Given that the PPAR family proteins serve an integrative role in controlling lipotoxicity [24], we can posit a simple mechanistic view of PPAR signaling:
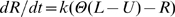
(1)where 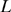 is the flux of lipid needing storage, 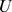 is the available capacity for lipid uptake, 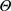 is the Heaviside function (0 for negative arguments and 1 for non-negative arguments), and 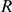 is the PPAR concentration. A growth model of adipose cell-size increase was proposed recently [15], in which adipose cells are recruited, grow in an intermediate size range, and then grow or decrease in size randomly due to lipid turnover at larger sizes. We can make a schematic discrete version of this continuous model. In our simple model, we describe adipocytes as being in one of three compartments, small, medium and large, with cell numbers denoted 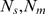, and 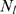 respectively. Thus, we take the available rate of lipid uptake to be 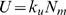, proportional to the number of medium sized adipocytes. An increase in 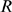 leads to the recruitment of new adipocytes that then develop further:
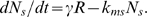
(2)The development of small adipocytes leads to an increase in size to an intermediate size:
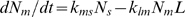
(3)and these intermediate-sized adipocytes take up available lipid and transition to the large adipocyte population:
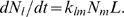
(4)Note that the rate at which medium-size adipocytes move to the large-size compartment is proportional to the flux of lipid needing storage, 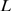. These equations exhibit periodic recruitment, Fig. 10, with a period that is dependent upon the flux of lipid needing storage. For example, with 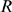 measured in units such that the maximal rate of increase of 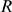 is numerically equal to 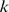, the rate of decay of 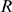, the set of parameters 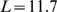 g/day, 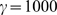 cell-number units/(day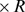-unit), 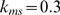/day, 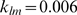/g, 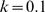/day, 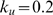 g/(cell-number units
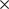
day) leads to a period of about 57 days. It should be possible to extend this simplified model to the continuous cell-size model proposed by [15]. Comparing the present data directly to the simplified ordinary differential equation model is difficult as it requires aggregating the detailed data into three bins with no clear biological motivation as to the locations of the bin boundaries. These boundary choices must then be considered fitting parameters, leading to a difficult optimization problem. Thus, combining our data with the schematic mathematical model, we predict that Zucker fatty rats on a reduced-fat or high-fat diet will exhibit periodic adipose tissue development with periods differing from those on a regular chow diet.

## Materials and Methods

### Surgical Biopsies

All procedures were carried out in accordance with NIH guidelines for rodent surgery and recovery. All animal work was conducted according to relevant national and international guidelines, approved by the National Institutes of Health, protocol # K027-DB-06. Two rats were subjected to the biopsies. Rats were anesthetized with isoflurane. The hair in the inguinal area was clipped and the skin cleaned three times with betadine surgical scrub, followed with an alcohol wipe. For adipogenesis studies in individual animals using surgical biopsies, the sampling procedure involves a small incision (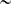
0.1 cm) in the skin just above the inguinal fat depot that lies immediately thereunder, dissection of a 15–20-mg sample of adipose tissue, and closing the incision with stainless steel clips. Clips were removed between 10–17 days. Significant pain and distress were not evident, but animals were closely observed for 2/3 h for potential problems. The inguinal fat depot surrounds the back side of the peritoneum, extending all the way from one hip to the other. Representative samples can be taken all across the fat depot and thus the incisions can be dispersed without giving up the required reproducibility. The biopsies were done on days 0, 2, 6, 9, 13, 23, 33, 57, 69, 86, 98, 134, 141, 150, 156 and 162, with data from rat 1 ending at day 150. Samples were thus taken all across the fat depot, alternating between the left and right sides, on both the abdominal surface and the back surface. Both rats were biopsied at the same location on any given day. The biopsies were done on days 0 (LA), 2 (RA), 6 (LA), 9 (RA), 13 (LA), 23 (RB), 33 (LB), 57 (RA), 69 (RB), 86 (LA), 98 ((RA), 134 (LB), 141 (RB), 150 (LB), 156 (RA), and 162 (RA), where L = left, R = right, A = abdomen, and B = back, with data from rat 1 ending at day 150. A biopsy done in the same general area where the clip from a previous biopsy was still in place (e.g. days 0 and 6) was spaced far enough away so as not to interfere with the healing of the previous wound.

### Measurement of Adipose Cell-Size

Adipose cell-size distribution was assessed using a Beckman Coulter Multisizer III as previously described [18]. Briefly, 15–20 mg of fat were immediately fixed in osmium tetroxide [25], [26] and incubated in a water bath at 37

C for 48 h. Adipose cell-size was then determined by a Beckman Coulter Multisizer III with a 400 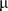
m aperture. The range of cell-sizes that can effectively be measured using this aperture is 20–240 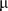
m. The instrument was set to count 6,000 particles, and the fixed-cell suspension was diluted so that coincident counting was 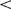
10%. After collection of pulse sizes, the data were expressed as particle diameters and displayed as histograms of counts against diameter using logarithmic bins and a logarithmic scale for the cell diameter. Cell-size distribution was measured four times from each biopsy, divided into two separate suspensions that were each counted twice, except for rat 1 at day 6, and rat 2 at days 13 and 86, with two cell-size distributions measured.

### Bayesian Analysis of Data

The aim of our analysis is to ascertain if the data exhibit temporal periodicity, and if so, to determine the characteristics of the periodic behavior. We need to generate a set of models that represent a fairly uniform set of possible periods in as large a range of periods as the data will allow.

The data consists of adipose cell-size distributions obtained at 16 time points, on days 0, 2, 6, 9, 13, 23, 33, 57, 69, 86, 98, 134, 141, 150, 156 and 162. Data from rat 1 end at day 150. Fractional differences of the average cell-size differences for days 33, 86 and 134 show a striking similarity, Fig. 3, and are distinct from the fractional differences for days 13 and 69, which are similar but not as similar to each other as those for days 33, 86 and 134. A useful depiction of the entire data-set (for rat 1) is in the form of a heat-map(Fig. 4), which conveys the present problem of determining if there is any periodicity, given limited views of the entire time course.

To analyze these data, we use a Bayesian model comparison. We suppose that the data can be modeled by a discrete model with 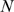 period-bins. Each period-bin corresponds to a duration of 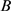 days. Such a model has a period 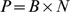 days. An additional variable that we need to generate concrete models is a choice of phase, 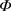, which is an integer between 0 and 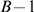. The phase is important since the data is sparse and irregularly distributed. Since we have a discrete set of experimental data points and a finite period-bin width, assigning biopsy days to model period-bins requires a choice of phase corresponding to where day 0 lies in the first period-bin:
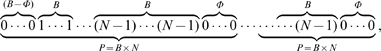
(5)with a certain number of repetitions of the basic period, 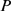, required to cover the entire time course of biopsies. The days of the experiment, numbered consecutively from day 0 through day 162, are aligned under this repetitive pattern and assigned the bin number directly above them. Thus, for each choice of 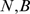 and phase, each biopsy day will fall into a period-bin number between 0 and 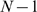. The biopsy days (0, 2, 6, 9, 13, 23, 33, 57, 69, 86, 98, 134, 141, 150, 156, 162) are mapped to a sequence of integers such as (0 1 1 2 2 3 5 1 2 4 6 3 4 5 6 0), which corresponds to the most likely model for rat 2 with 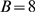 days, 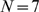 period-bins, and 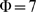 days, and therefore, a period of 56 days. For rat 1, the most likely model sequence is two time points shorter, 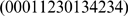, with 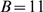 days, 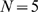 period-bins and 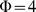. The model of no period has one bin only, and thus corresponds to a sequence 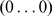.

We took the number of period-bins to be between 2 and 10, and the period-bin size to be between 5 days and 50 days. This leads to a large set of possible models, 579 models for rat 1 and 683 models for rat 2. Not all these models test periodicity, nor are they all testable with our data. We prune the list of models based on the following criteria.

The periodicity in the model must be testable using the data. We accept only models in which the period-bin assignments are such that every period-bin has at least some biopsy days assigned to it that are not all contiguous in the sequence of experiment days; for example, an assignment of period-bins (0 0 0 0 1 1 2 3 0 2 2 3) is not acceptable since it has period-bin number 1 assigned only to contiguous days, while (0 0 0 0 1 1 2 0 1 2 2 1) is acceptable since all period-bin numbers appear in at least two non-contiguous locations in the time sequence. This ensures that every period-bin in a period has at least two non-contiguous experimental days assigned to it, and therefore that if the data values associated with a period-bin are approximately the same, this is not merely the result of continuity, since the period-bin values arise from non-contiguous locations in the experimental data.

With an experiment that covered 163 days, it is mathematically difficult to discern periods larger than about 100 days. With intervals between biopsy days ranging from 3 days to 36 days, it is not feasible to detect periods below about 30 days. We reject all models with periods that do not meet these criteria. The upper limit of 100 days may seem overly ambitious but we want to err on the side of a larger set of models and let the Bayesian model selection reject these models as unlikely.

Of the resulting reduced set of models, we note that the models do not all correspond to a unique period, since the bin assignments are discrete choices with non-zero bin-widths. However, the largest ambiguity in the period in any selected model is less than 6 days, and the mean period ambiguity over all the 358 discrete models for rat 1 is 0.5 days, and over all the 487 discrete models for rat 2 is 0.4 days. None of the ambiguous models is within the 10 most likely models for either rat so we do not discuss them further.

The data for each time point consists of 80 cell-size bins, logarithmically binned with increasing cell-size bins containing an increasing range of adipose cell-sizes. All cell counts in each cell-size bin are independent of all other bins, except for the overall normalization of the cell-size distribution. We thus evaluate the probability of each cell-size bin exhibiting a periodic behavior independently of other cell-size bins.

For each cell-size bin, the measured probability on days corresponding to the same period-bin should be the same up to measurement uncertainty. Therefore we model the log likelihood of counts for a given cell-size bin, 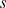, and a given period-bin, 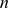, as the logarithm of the lognormal probability density (up to an irrelevant constant)
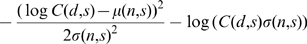
(6)where the parameters 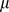 and 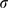 depend on the period-bin number 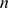, and the counts 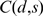 depend on the biopsy day 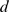 that was mapped to period-bin number 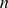 in the model; both the parameters, 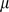 and 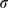, and the counts 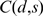 depend on the cell-size bin, 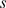. Summing this likelihood over all days that correspond to a particular period-bin, and then summing over period-bin numbers, gives us the log likelihood for the data given the counts at cell-size bin 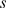. We marginalize over 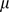 and 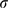 using a parallel tempered Monte Carlo procedure given by Gregory [27], with uninformative priors for 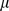 and 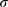. We initialize the Monte Carlo procedure by setting the initial value 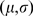 to the (mean, standard deviation) of the logarithm of the counts for the days assigned to that period-bin.

The Monte Carlo carried out equilibration for 20000 steps (with a mean fractional step size of 0.1) and then stored for the next 2000 steps (with a mean fractional step size of 0.02). During the equilibration time, the parallel tempering procedure switched temperatures with a probability of 0.05, corresponding to about once every twenty steps. After equilibration, the parallel tempering steps were all started from the same initial value, the maximum likelihood value found during the equilibration phase of the Monte Carlo procedure, and did not undergo any further switching between temperatures to ensure that the integration-over-temperatures interpretation of parallel tempering was valid.

Having obtained the likelihood of a particular periodic model given a cell-size bin, we summed over all cell-size bins, excluding one cell-size bin to take the normalization constraint into account, to obtain our final results.

All analysis was performed in XLispStat [28].
