# Correction: Waves of Adipose Tissue Growth in the Genetically Obese Zucker Fatty Rat

**DOI:** 10.1371/annotation/937ee9b8-124d-4a08-91c1-4f0f9cf1a742

**Published:** 2010-04-09

**Authors:** Jennifer MacKellar, Samuel W. Cushman, Vipul Periwal

This article has been republished because Figures 1-4 were originally published in the incorrect order. 

